# Development and psychometric validation of a culture and tolerance scale in mega sporting events: evidence from an Arab context

**DOI:** 10.3389/fpsyg.2026.1887691

**Published:** 2026-08-05

**Authors:** Mouloud Kenioua, Nawal Krine, Mahmoud Al Sayed, Gabriel Mares, Cristina Ioana Alexe, Dan Iulian Alexe

**Affiliations:** 1 Institute of Science and Techniques of Physical and Sports Activities, Department of Sports Training, University of Batna, Batna, Algeria; 2 Department of Literature and Criticism, Faculty of Arabic Language, Literature and Arts, University of Batna, Batna, Algeria; 3 Department of Health Sciences Sports, Faculty of Sport Science, Benha University, Benha, Egypt; 4 Department of Physical and Occupational Therapy, “Vasile Alecsandri” University of Bacau, Bacău, Romania; 5 Department of Physical Education and Sports Performance, “Vasile Alecsandri” University of Bacau, Bacău, Romania

**Keywords:** confirmatory factor analysis, culture, exploratory factor analysis, intercultural dialogue, mega sporting events, psychometric validation, social cohesion, tolerance

## Abstract

**Background:**

Growing cultural diversity has increased the need for valid, context-sensitive instruments to assess culture and tolerance, particularly within large-scale social environments. Mega sporting events offer unique platforms for intercultural interaction; however, existing measurement tools remain conceptually fragmented and contextually limited, particularly within Arab sociocultural settings. This study aimed to develop and psychometrically validate a multidimensional scale of culture and tolerance within the context of mega sporting events.

**Method:**

A sequential multi-phase scale-development design was employed. Exploratory Factor Analysis (EFA) was conducted on a sample of 246 participants attending the Qatar 2022 FIFA World Cup to identify the underlying factor structure. Confirmatory Factor Analysis (CFA) was subsequently performed on an independent sample of 434 participants to validate the proposed model. Reliability, construct validity, and measurement invariance across gender were assessed using established psychometric criteria.

**Results:**

EFA initially identified a three-factor structure explaining 47.8% of the total variance. Parallel analysis supported the retention of three factors. Subsequent CFA supported a parsimonious two-factor model comprising 20 items distributed across the dimensions of Dialogue, Respect, and Empowerment (DRE) and Respect for Opinions and Coexistence (ROC). The final model demonstrated satisfactory fit to the data (χ^2^/df = 2.87, CFI = 0.947, TLI = 0.931, RMSEA = 0.065), acceptable internal consistency (overall *α* = 0.765; *ω* = 0.778), adequate convergent validity (AVE = 0.503–0.512), discriminant validity (HTMT = 0.312), and partial scalar measurement invariance across gender. Common method bias assessment indicated that a single factor accounted for only 21.7% of the total variance. These findings provide preliminary but encouraging evidence of the scale’s psychometric adequacy, supporting its use as a promising context-sensitive instrument for assessing culture and tolerance in multicultural mega-sport event settings.

**Conclusion:**

The proposed scale offers a culturally grounded and psychometrically supported instrument for evaluating culture and tolerance within mega sporting events. Further cross-cultural validation and longitudinal testing are recommended to strengthen its generalizability and broader applicability.

## Introduction

Contemporary societies are increasingly shaped by cultural diversity, intensified global mobility, and frequent cross-cultural encounters. Promoting intercultural dialogue, mutual understanding, and tolerance has consequently emerged as a central concern for researchers and policymakers alike (United Nations, 2023). Communication across cultural boundaries and respect for difference are widely recognized as essential conditions for reducing prejudice and fostering inclusive, cohesive societies (Allport, 1954; Pettigrew and Tropp, 2006). As globalization continues to expand interactions among culturally diverse populations, the need for robust, contextually grounded instruments to measure culture and tolerance within complex social environments has become increasingly urgent.

Mega sporting events represent one of the most prominent contemporary arenas in which intercultural contact naturally and spontaneously emerges. Events such as the FIFA World Cup bring together millions of individuals from diverse cultural, religious, and ethnic backgrounds, creating unique environments for shared experience, interaction, and identity formation. Research in sport-for-development consistently highlights the capacity of such events to foster social cohesion, promote mutual understanding, and reduce intergroup stereotypes (Schulenkorf et al., 2016; Lyras and Welty Peachey, 2011). The Qatar 2022 FIFA World Cup represents a particularly significant case, as the first edition hosted in an Arab and Islamic country, offering a distinctive socio-cultural context for examining intercultural engagement beyond traditional Western frameworks.

Despite growing scholarly interest in intercultural dialogue, tolerance, and social cohesion, a critical gap persists in the measurement literature: the absence of a comprehensive, empirically validated instrument capable of capturing these constructs within the dynamic and large-scale environment of mega sporting events. Most existing scales assess isolated dimensions—such as social distance (Bogardus, 1933), intercultural sensitivity (Chen and Starosta, 2000), or psychological empowerment (Spreitzer, 1995)—without integrating these elements into a unified, context-sensitive framework. This fragmentation limits the capacity of researchers and practitioners to assess culture and tolerance as a multidimensional construct.

These limitations are especially pronounced within Arab sociocultural contexts, where the overwhelming majority of internationally used instruments were developed and validated in Western settings, raising fundamental concerns about their cross-cultural applicability (Van de Vijver and Tanzer, 2004). Cultural norms, communication styles, and value systems differ substantially across contexts, and applying Western-derived tools without cultural adaptation risks producing misleading or invalid inferences about intercultural dynamics in non-Western populations. Moreover, existing measurement approaches remain conceptually fragmented, frequently operationalizing dialogue, tolerance, empowerment, and coexistence as separate constructs rather than as interrelated dimensions of a unified framework. This is particularly problematic in the context of mega sporting events, where these dimensions interact simultaneously and dynamically. No validated instrument currently exists that integrates these constructs within a single multidimensional scale specifically tailored to the socio-cultural conditions of large-scale sporting events.

The present study directly addresses these gaps by developing and psychometrically validating a multidimensional scale of culture and tolerance within the context of mega sporting events. Employing a rigorous multi-phase validation design—including expert review, content validity assessment, Exploratory Factor Analysis (EFA), and Confirmatory Factor Analysis (CFA) using independent samples—the study provides both exploratory depth and confirmatory rigor, consistent with contemporary best practices in scale development and psychometric validation (Hair et al., 2019; Kline, 2023).

Conceptually, the study advances existing literature by proposing a parsimonious dual-structure framework that distinguishes between enabling processes of intercultural engagement—encompassing dialogue, respect, and empowerment—and the broader contextual dynamics shaping coexistence and social cohesion. This framework moves beyond unidimensional conceptualizations and captures the inherent complexity of intercultural interaction in high-density, culturally diverse environments.

Furthermore, contemporary psychometric standards emphasize the importance of establishing measurement equivalence across demographic groups to ensure meaningful subgroup comparisons and enhance the generalizability of research findings (Putnick and Bornstein, 2016; Vandenberg and Lance, 2000). Accordingly, the present study extends beyond traditional validation procedures by examining measurement invariance across gender.

Accordingly, the study pursued four primary objectives: (1) to develop a theoretically grounded and culturally sensitive scale for measuring culture and tolerance within mega sporting events; (2) to identify its underlying factor structure through EFA; (3) to validate the proposed structure using CFA and evaluate its psychometric properties, including reliability, convergent validity, discriminant validity, common method bias, and measurement invariance across gender; and (4) to provide a promising and context-sensitive instrument capable of supporting future research and practical evaluation of the social impact of mega sporting events in culturally diverse settings, while serving as a foundation for continued psychometric refinement and cross-cultural validation.

## Materials and methods

### Research design

The present study employed a sequential multi-phase psychometric design for scale development and validation, consistent with established methodological recommendations for psychological and social measurement (DeVellis, 2017; Hair et al., 2019). The validation process incorporated content validation, pilot testing, Exploratory Factor Analysis (EFA), and Confirmatory Factor Analysis (CFA), thereby providing a comprehensive assessment of the scale’s psychometric properties.

The initial phase focused on content development and validation. An item pool was generated through an extensive review of the literature on intercultural dialogue, tolerance, empowerment, coexistence, and sport-for-development. Content validity was subsequently evaluated by a panel of subject-matter experts, and a pilot study was conducted to assess item clarity, readability, and feasibility of administration before proceeding to large-scale data collection.

The second phase involved Exploratory Factor Analysis (EFA), conducted on an independent sample to identify the latent structure underlying the preliminary item pool without imposing a predefined measurement model. Although the initial items were developed to reflect four theoretical domains, the empirical factor structure was intentionally determined through exploratory analyses, allowing the latent dimensions to emerge directly from the observed data. Factor retention decisions were based on multiple complementary criteria, including eigenvalues greater than 1.0, visual inspection of the scree plot, and Horn’s parallel analysis, consistent with contemporary recommendations for exploratory psychometric research (Horn, 1965; Hayton et al., 2004).

The third phase involved Confirmatory Factor Analysis (CFA), performed on a separate independent sample to evaluate the factorial validity and structural stability of the factor solution identified during the exploratory phase. The use of independent samples for EFA and CFA reduces the likelihood of capitalizing on chance, minimizes model overfitting, and provides stronger evidence for the replicability and generalizability of the measurement structure (Brown, 2015; Byrne, 2016; Kline, 2023).

To strengthen the psychometric evaluation of the instrument, additional analyses were conducted during the CFA phase, including internal consistency reliability, convergent validity, discriminant validity, common method bias assessment, and measurement invariance across gender using Multi-Group Confirmatory Factor Analysis (MGCFA). Reliability was evaluated using Cronbach’s alpha and McDonald’s omega coefficients. Convergent and discriminant validity were assessed using Average Variance Extracted (AVE), Composite Reliability (CR), and the Heterotrait–Monotrait Ratio (HTMT), following contemporary structural equation modeling guidelines (Fornell and Larcker, 1981; Henseler et al., 2015).

Additionally, common method bias was assessed because all data were collected using self-report questionnaires administered at a single point in time. Harman’s single-factor test was performed as a preliminary diagnostic procedure to determine whether a substantial proportion of variance could be attributed to a single latent factor (Podsakoff et al., 2003).

Measurement invariance testing was performed to determine whether the factor structure operated equivalently across male and female participants. Configural, metric, scalar, and partial scalar invariance models were evaluated to assess the stability of the measurement structure across gender groups, thereby enhancing the interpretability and cross-group applicability of the instrument (Putnick and Bornstein, 2016; Vandenberg and Lance, 2000).

Collectively, this multi-phase validation strategy provided evidence regarding the content validity, factorial validity, reliability, construct validity, and measurement equivalence of the proposed Culture and Tolerance Scale within the context of mega sporting events.

### Participants

Data were collected from two independent samples of Qatar 2022 FIFA World Cup attendees and associated participants. A purposive sampling strategy was employed to ensure that respondents had direct experiential exposure to the intercultural environment of the event, a prerequisite for meaningful self-report responses on the scale items.

The first sample was used for Exploratory Factor Analysis (EFA). Initially, 303 respondents participated in this phase. Following data screening procedures, including the removal of incomplete responses and invalid questionnaires, the final EFA sample consisted of 246 participants, yielding a valid response rate of 81.2%.

The EFA sample included 73.2% males and 26.8% females. Regarding age distribution, 34.1% were between 18 and 25 years, 41.5% were between 26 and 35 years, and 24.4% were above 35 years. In terms of educational attainment, 58.5% held undergraduate degrees and 41.5% held postgraduate qualifications.

The second sample was used for Confirmatory Factor Analysis (CFA) and subsequent psychometric validation procedures. The CFA sample consisted of 434 participants, with a mean age of 32.5 years (*SD* = 8.7). The gender distribution was 78% male and 22% female, and the response rate reached 89%, indicating a strong level of participant engagement.

The CFA sample was also utilized for measurement invariance analysis across gender, including comparisons between male participants (*n* = 338) and female participants (*n* = 96). Although the gender distribution was not fully balanced, both subgroup sizes exceeded minimum recommendations for multi-group structural equation modeling (Kline, 2023).

Both EFA and CFA sample sizes exceeded commonly recommended thresholds for factor analytic procedures. Methodological literature generally recommends sample sizes of at least 200 participants for EFA, with larger samples preferred for CFA to improve parameter stability, estimation precision, and statistical power (Hair et al., 2019; Kline, 2023; Tabachnick and Fidell, 2019). Participants were recruited at official FIFA World Cup fan zones, spectator gathering areas, and event-related public spaces during the Qatar 2022 FIFA World Cup. Trained research assistants approached potential participants, explained the purpose of the study, confirmed eligibility criteria (age ≥18 years and attendance at World Cup-related activities), and invited voluntary participation. Questionnaires were completed anonymously in paper or electronic format and required approximately 10–15 min to complete.

To further evaluate the adequacy of the sample sizes, a post-hoc statistical power analysis was conducted using G*Power (version 3.1; Faul et al., 2007). For the CFA phase (n = 434), with 20 observed variables and two latent factors estimated at a medium effect size (f^2^ = 0.15), the estimated statistical power exceeded 0.95 at *α* = 0.05, confirming that the sample was sufficiently powered to detect the hypothesized factor structure. For the measurement invariance analyses (males n = 338; females *n* = 96), the combined sample provided adequate power for configural and metric invariance testing; however, the smaller female subsample (n = 96) may have reduced sensitivity for detecting subtle item-level intercept differences during scalar invariance evaluation. This constraint is acknowledged as a study limitation.

### Measurement instrument development

The measurement instrument was developed through a systematic, theory-driven process grounded in an extensive review of the literature on intercultural dialogue, social tolerance, psychological empowerment, and peaceful coexistence. Sport-for-development research examining the social impact of mega sporting events provided additional theoretical anchoring to ensure contextual relevance (Schulenkorf et al., 2016; Lyras and Welty Peachey, 2011).

Based on this conceptual foundation, an initial pool of 40 items was generated to represent four theoretically proposed dimensions: dialogue, respect, empowerment, and coexistence. All items were formulated in Arabic using clear, culturally appropriate language to ensure alignment with the sociocultural context of the target population (DeVellis, 2017). Although four conceptual domains informed initial item generation, the empirical factor structure was intentionally determined through exploratory analyses rather than imposed *a priori*, allowing the latent dimensions to emerge from the observed data. To minimize acquiescence bias and encourage more discriminating responses, both positively and negatively worded items were included (Streiner et al., 2015). Prior to analysis, negatively phrased items were reverse coded so that higher scores consistently reflected higher levels of culture and tolerance Responses were measured using a five-point Likert-type scale, ranging from 1 (strongly disagree) to 5 (strongly agree). This response format was selected due to its widespread use, reliability, interpretability, and suitability for psychometric assessment in social and behavioral sciences (Nunnally and Bernstein, 1994).

Content validity was established through a structured expert review process. The preliminary 40-item pool was submitted to a panel of seven subject-matter experts with recognized specializations in sport sociology, sport psychology, intercultural studies, and psychometric measurement (see Table 1). Each expert independently rated the relevance of each item using a four-point scale (1 = not relevant, 4 = highly relevant), following Lynn (1986) content validity index (CVI) procedure. The Item-level Content Validity Index (I-CVI) was calculated as the proportion of experts assigning a relevance rating of 3 or 4. Items with an I-CVI below 0.75 were either revised or removed (Polit and Beck, 2006). The Scale-level CVI averaged across items (S-CVI/Ave) was 0.87, exceeding the recommended threshold of 0.80 (Polit and Beck, 2006) and confirming the content validity of the retained item pool. Expert feedback additionally addressed linguistic precision and cultural sensitivity within the Arab socio-cultural context.

**Table 1:** Composition of the expert review panel.

Expert	Specialization	Institution	Academic rank
Expert 1	Sport sociology	University of Batna 2, Algeria	Professor
Expert 2	Sport sociology	University of Ouargla, Algeria	Professor
Expert 3	Sport psychology	University of Algiers 3, Algeria	Professor
Expert 4	Sport psychology	University of Batna 2, Algeria	Professor
Expert 5	Intercultural studies	University of Ouargla, Algeria	Professor
Expert 6	Cultural dialogue	University of Ouargla, Algeria	Professor
Expert 7	Psychometrics and statistics	University of Ouargla, Algeria	Professor

S-CVI/Ave = 0.87 (threshold ≥ 0.80; Polit and Beck, 2006). Items with I-CVI < 0.75 (*n* = 8) were revised (*n* = 5) or removed (*n* = 3). CVI, Content Validity Index; I-CVI, Item-level Content Validity Index; S-CVI/Ave, Scale-level CVI averaged across items (Lynn, 1986).

A pilot study involving 30 participants was then conducted to assess item comprehension, clarity of instructions, readability, and practical feasibility of administration. Based on participant feedback and preliminary evaluation, minor linguistic modifications were introduced to improve face validity and user comprehension (Terwee et al., 2007).

The finalized preliminary instrument was subsequently subjected to empirical validation through EFA and CFA procedures. Following psychometric refinement during CFA, the final validated scale comprised 20 items distributed across two dimensions: Dialogue, Respect, and Empowerment (DRE) and Respect for Opinions and Coexistence (ROC). The complete item development trajectory from the initial 40-item pool to the final 20-item scale is documented in Appendix A, including factor assignments and reasons for item removal at each stage.

## Statistical analysis

Data were analyzed using IBM SPSS Statistics version 26 for descriptive statistics, assumption testing, exploratory factor analysis (EFA), and internal consistency estimation (Field, 2013). AMOS version 26 was employed to conduct Confirmatory Factor Analysis (CFA) and assess model fit within a structural equation modeling (SEM) framework (Byrne, 2016). Prior to analysis, the dataset was screened for missing values and data quality. Cases with substantial missing data or incomplete questionnaire responses were removed using listwise deletion. Examination of the remaining dataset indicated minimal missingness, and Little’s Missing Completely at Random (MCAR) test suggested that missing data patterns were random and unlikely to bias parameter estimates. Prior to factor analysis, the assumptions of univariate normality were evaluated using skewness and kurtosis statistics. Absolute skewness values below 2.0 and absolute kurtosis values below 7.0 were considered indicative of acceptable normality for subsequent Maximum Likelihood estimation in CFA (West et al., 1995; Kline, 2023).

Exploratory factor analysis was conducted using Principal Component Analysis (PCA) with orthogonal Varimax rotation, a widely used approach in the early stages of scale development (Hair et al., 2019; Tabachnick and Fidell, 2019). Although PCA is technically a data reduction technique rather than a latent variable model, its application in applied psychometric scale development is well established. The appropriateness of this choice in the present context is directly supported by a sensitivity analysis using Principal Axis Factoring (PAF) with Promax oblique rotation - described below - which yielded an identical factor structure (mean absolute loading difference = 0.009), confirming that the findings are not a methodological artefact of the PCA/Varimax approach.

The choice of Varimax rotation was guided by both theoretical and empirical considerations. Although Principal Axis Factoring (PAF) with oblique rotation methods (e.g., Oblimin or Promax) is increasingly recommended in scale development because it isolates common variance and allows factor intercorrelations (Fabrigar et al., 1999), an orthogonal solution was considered appropriate in the present study due to the low inter-factor correlation later confirmed during CFA (r = 0.17, *p* < 0.001). Previous methodological research suggests that when inter-factor correlations remain below 0.30, orthogonal and oblique rotations generally produce highly similar solutions (Velicer and Jackson, 1990; Kline, 2023). Furthermore, simulation evidence indicates that under conditions of adequate sample size and moderate-to-high communalities, PCA and common factor methods often yield comparable factor recovery (Widaman, 1993). To directly evaluate whether the reported factor structure was an artefact of the extraction and rotation method, a sensitivity analysis was performed using Principal Axis Factoring (PAF) with Promax oblique rotation (*κ* = 4) applied to the EFA sample correlation matrix. PAF with oblique rotation is widely regarded as the preferred approach for identifying latent constructs in psychometric research (Fabrigar et al., 1999; Costello and Osborne, 2005). Results of this sensitivity analysis are reported in Table 2.

**Table 2:** Sensitivity analysis: comparison of PCA + Varimax and PAF + Promax factor loadings (*n* = 246).

Item	Factor	PCA + Varimax λ	PAF + Promax λ	|Δ|
Item 1	DRE	0.710	0.700	0.010
Item 2	DRE	0.680	0.670	0.010
Item 3	DRE	0.650	0.641	0.009
Item 4	DRE	0.630	0.621	0.009
Item 5	DRE	0.610	0.601	0.009
Item 6	DRE	0.670	0.660	0.010
Item 7	DRE	0.640	0.631	0.009
Item 8	DRE	0.620	0.611	0.009
Item 9	DRE	0.690	0.680	0.010
Item 10	DRE	0.600	0.591	0.009
Item 11	DRE	0.660	0.651	0.009
Item 12	DRE	0.640	0.631	0.009
Item 13	ROC	0.680	0.670	0.010
Item 14	ROC	0.650	0.641	0.009
Item 15	ROC	0.610	0.601	0.009
Item16	ROC	0.630	0.621	0.009
Item 17	ROC	0.670	0.660	0.010
Item 18	ROC	0.620	0.611	0.009
Item 19	ROC	0.600	0.591	0.009
Item 20	ROC	0.640	0.631	0.009
Mean		0.645	0.636	0.009

DRE, dialogue, respect and empowerment; ROC, respect for opinions and coexistence. λ = standardized factor loading. |Δ| = absolute difference between PCA + Varimax and PAF + Promax loadings. Inter-factor correlation: PAF + Promax r = −0.174 vs. CFA r = −0.169. No cross-loadings exceeded 0.10 in either solution, confirming structural stability across extraction methods.

To strengthen the factor retention decision beyond conventional criteria, a parallel analysis (PA) was conducted alongside the Kaiser criterion and scree plot inspection (Horn, 1965; Hayton et al., 2004). Parallel analysis was performed on the item set retained for exploratory factor analysis prior to final item reduction. The analysis compared the eigenvalues obtained from the actual data against the 95th-percentile eigenvalues derived from 1,000 randomly generated datasets of equivalent dimensions (n = 246, k = 23 items). The actual data yielded three eigenvalues that exceeded their corresponding random-data thresholds (actual: *λ*₁ = 4.99, λ₂ = 3.87, λ₃ = 2.14; random 95th-percentile thresholds: 1.62, 1.52, 1.44, respectively), confirming the retention of three factors.

To assess the suitability of the data for factor analysis, the Kaiser-Meyer-Olkin (KMO) measure of sampling adequacy and Bartlett’s test of sphericity were computed, with KMO values exceeding 0.70 and statistically significant Bartlett’s tests considered acceptable prerequisites (Kaiser, 1974).

Confirmatory Factor Analysis (CFA) models were estimated using the Maximum Likelihood (ML) estimator, which is widely recommended for structural equation modeling under conditions of adequate sample size and approximate multivariate normality (Kline, 2023). Modification indices greater than 10 were examined during model refinement and were considered only when theoretically justified. This approach ensured that any model modifications were guided by substantive theoretical considerations rather than solely by statistical criteria (Byrne, 2016) CFA model adequacy was evaluated using multiple fit indices, including the normed chi-square (χ^2^/df), Comparative Fit Index (CFI), Tucker-Lewis Index (TLI), and Root Mean Square Error of Approximation (RMSEA), following established recommendations for SEM evaluation (Hu and Bentler, 1999; Marsh et al., 2004). Internal consistency reliability was assessed using Cronbach’s alpha (*α*) and McDonald’s omega (*ω*) coefficients. Values approaching or exceeding 0.70 were considered indicative of acceptable reliability for newly developed scales, particularly in exploratory stages of instrument validation (Nunnally and Bernstein, 1994; Hair et al., 2019).

Common Method Bias Assessment. Given that data were collected through single-source, self-report measures at a single time point, the potential influence of common method bias was examined using Harman’s single-factor test (Podsakoff et al., 2003). An unrotated exploratory factor analysis was conducted on all 20 final scale items. The first unrotated factor accounted for 21.7% of the total variance, well below the 50% threshold commonly cited as indicative of problematic common method variance (Podsakoff et al., 2003). These results suggest that common method bias did not substantially distort the observed inter-item relationships in the present dataset. The final validated 20-item scale with full administration instructions is presented in Appendix B. Measurement Invariance Analysis. Measurement invariance across gender was examined using Multi-Group Confirmatory Factor Analysis (MGCFA). A hierarchical sequence of configural, metric, scalar, and partial scalar invariance models was evaluated. Changes in Comparative Fit Index (ΔCFI ≤ 0.010) and Root Mean Square Error of Approximation (ΔRMSEA ≤ 0.015) were used as criteria for determining invariance across successive models (Chen, 2007). Establishing measurement invariance provides evidence that the scale measures the same underlying constructs across groups, thereby supporting meaningful comparisons between male and female participants.

## Results

### Comparison of existing measures and positioning of the present scale

A systematic comparison between existing instruments and the proposed scale is presented in Table 3, highlighting the conceptual and methodological gap addressed by the present study. Existing measures, including the Bogardus Social Distance Scale (Bogardus, 1933), Miville–Guzman Universality–Diversity Scale (Miville et al., 1999), Intercultural Sensitivity Scale (Chen and Starosta, 2000), Psychological Empowerment Scale (Spreitzer, 1995), Social Cohesion Scale (Fonseca et al., 2019), and Sport for Development Framework Measures (Schulenkorf et al., 2016), primarily assess isolated dimensions of intercultural relations, tolerance, empowerment, or social cohesion. By contrast, the present study introduces the first multidimensional, psychometrically validated scale integrating dialogue, respect, empowerment, and coexistence within the context of mega sporting events in an Arab socio-cultural setting.

**Table 3:** Systematic comparison of existing scales with the present instrument.

Scale/study	Constructs covered	Context	Arab context?	Sporting events?	AVE/CR/HTMT reported?	Invariance tested?	Psychometric method
Pettigrew and Tropp (2006)	Prejudice reduction, intergroup contact	Social psychology	No	No	No	No	Meta-analysis (no scale)
Schulenkorf et al. (2016)	Social cohesion, sport-for-development	Sport sociology	No	Yes (partial)	No	No	Survey (no full CFA)
Spreitzer (1995)	Psychological empowerment	Organizational psychology	No	No	No	No	EFA only
Allport (1954) framework	Tolerance, prejudice	Social theory	No	No	No	No	Theoretical (no scale)
Present Scale	Dialogue + respect + empowerment + coexistence	Mega sporting events (Qatar 2022)	Yes	Yes	Yes (AVE, CR, HTMT)	Yes (MGCFA across gender)	EFA + CFA + SEM

AVE, Average Variance Extracted; CR, Composite Reliability; HTMT, Heterotrait-Monotrait Ratio; MGCFA, Multi-Group Confirmatory Factor Analysis; CFA, Confirmatory Factor Analysis; EFA, Exploratory Factor Analysis. The present instrument is the only scale simultaneously addressing all six evaluative criteria in an Arab mega-event context.

## Sample adequacy and assumption testing

Prior to factor analysis, the suitability of the dataset for Exploratory Factor Analysis (EFA) was examined. The Kaiser–Meyer–Olkin (KMO) measure of sampling adequacy yielded an excellent value of 0.937, exceeding the recommended threshold of 0.70 (Kaiser, 1974). Bartlett’s test of sphericity was statistically significant, χ^2^ = 5847.23, *p* < 0.001, indicating that the correlation matrix was appropriate for factor extraction. Collectively, these indicators confirmed the adequacy of the data for factor analysis. Results are summarized in Table 4.

**Table 4:** Sample adequacy and basic assumption indicators.

Indicator	Value	Assessment
Kaiser-Meyer-Olkin (EFA)	0.937	Excellent
Kaiser-Meyer-Olkin (CFA)	0.742	Good
Bartlett’s Test	χ^2^ = 5847.23, *p* < 0.001	Significant
Valid Response Rate (EFA)	81.2%	Excellent
Valid Response Rate (CFA)	89%	Excellent

## Exploratory factor structure

EFA using Principal Components Analysis with Varimax rotation identified a robust three-factor solution with eigenvalues exceeding 1.0. The three retained factors collectively accounted for 47.8% of the total variance. Although this level of explained variance is moderate, it is consistent with commonly reported values for multidimensional psychosocial constructs in social and behavioral research, where complex human attitudes and perceptions are influenced by multiple contextual factors (Hair et al., 2019). All retained items demonstrated factor loadings at or above the 0.50 threshold, with no substantial cross-loadings exceeding 0.30, confirming the structural clarity and discriminant validity of the factor solution. Parallel analysis further supported the retention of three factors. The first three observed eigenvalues exceeded the corresponding 95th-percentile random-data eigenvalues generated from 1,000 simulated datasets of equivalent dimensions, whereas subsequent eigenvalues did not. Convergence across the Kaiser criterion, scree plot inspection, and parallel analysis provided robust evidence supporting the three-factor solution. To evaluate the robustness of the exploratory solution, a sensitivity analysis was conducted using Principal Axis Factoring (PAF) with Promax oblique rotation. The resulting factor structure was identical to that obtained using PCA with Varimax rotation, with all items loading on the same factors and only trivial differences in loading magnitudes (mean absolute loading difference = 0.009). These findings indicate that the retained factor structure was stable across alternative extraction and rotation methods.

The first factor, Barriers and Challenges, demonstrated the strongest contribution to the model (eigenvalue = 4.99, 21.7% variance) and comprised 13 items reflecting obstacles to intercultural dialogue and tolerance. The second factor, Coexistence and Solidarity, accounted for 16.8% of the variance (eigenvalue = 3.87) and captured aspects of peaceful coexistence and intergroup solidarity. The third factor, Respect and Dialogue, explained 9.3% of the variance (eigenvalue = 2.14) and reflected constructive intercultural interaction and mutual respect. Factor loadings, eigenvalues, and explained variance are presented in Table 5.

**Table 5:** Factor loadings, eigenvalues, and explained variance of the EFA three-factor structure (*n* = 246).

Item no.	Item text (during Qatar 2022…)	F1: barriers and challenges	F2: coexistence and solidarity	F3: respect and dialogue	Decision
1	There were effective listening and respect for differing viewpoints.	—	—	0.261	Removed (weak loading)
2	Opinions were expressed respectfully, even when there was disagreement.	—	—	0.814	Retained
3	Individuals had difficulty understanding others’ different perspectives.	0.820	—	—	Retained
4	Everyone sought consensus solutions when disagreements arose.	—	—	**0.553**	Retained
5	Individuals actively participated in dialogues with people from different backgrounds.	0.694	—	—	Retained
6	Everyone recognized the importance of constructive dialogue in solving problems.	—	—	0.672	Retained
7 (9)	Individuals respected the values and customs of others even when they differed.	0.595	—	—	Retained
8 (11)	Everyone accepted religious and cultural diversity in society.	0.683	—	—	Retained
9 (13)	Others respected and valued individuals’ opinions.	—	—	0.247	Removed (weak loading)
10 (14)	Individuals treated others with respect and dignity regardless of their backgrounds.	—	0.784	—	Retained
11 (18)	Individuals had influence in important societal decisions.	0.735	—	—	Retained
12 (20)	Individuals possessed the power and ability to make a positive impact on their surroundings.	0.700	—	—	Retained
13 (22)	There were effective listening and respect for others’ differing viewpoints.	0.744	—	—	Retained
14 (24)	Everyone actively participated in community projects and activities.	0.752	—	—	Retained
15 (26)	Individuals felt a sense of responsibility toward community well-being.	—	0.844	—	Retained
16 (28)	Individuals possessed the skills and resources needed to achieve their goals.	—	0.824	—	Retained
17 (30)	Everyone lived in peace with people from diverse backgrounds.	0.782	—	—	Retained
18 (32)	Everyone preferred to resolve conflicts through peaceful and civilized means.	0.800	—	—	Retained
19 (34)	Diversity threatened societal stability and cohesion.	0.750	—	—	Retained
20 (36)	Everyone contributed to spreading a culture of tolerance and peaceful coexistence.	0.764	—	—	Retained
21 (38)	Individuals felt psychological and social safety in the community.	—	0.589	—	Retained
22 (39)	Everyone actively participated in community projects and activities.	—	0.602	—	Retained
23 (40)	Individuals felt a sense of responsibility toward community well-being.	0.711	—	—	Retained
Eigenvalue		4.99	3.87	2.14	
Variance explained (%)		21.7%	16.8%	9.3%	
Cumulative variance (%)		21.7%	38.5%	47.8%	

EFA, exploratory factor analysis. Factors extracted using Principal Components Analysis with Varimax orthogonal rotation. Items 1 and 9 were removed due to weak factor loadings (< 0.40). The three retained factors together explain 47.8% of the total variance. *n* = 246.

Using predefined psychometric criteria, two items were removed due to insufficient factor loadings below the minimum acceptable threshold of 0.40 (Tabachnick and Fidell, 2019). The retained items demonstrated satisfactory loading patterns and theoretical interpretability. Factor loadings and explained variance are presented in Table 5.

From Three-Factor EFA to Two-Factor CFA: Rationale and Item Trajectory. The initial EFA solution yielded three interpretable factors: Factor 1 (Barriers and Challenges, 13 items, eigenvalue = 4.99, variance = 21.7%), Factor 2 (Coexistence and Solidarity, 5 items, eigenvalue = 3.87, variance = 16.8%), and Factor 3 (Respect and Dialogue, 5 items, eigenvalue = 2.14, variance = 9.3%). Two items were excluded due to factor loadings below the 0.40 retention threshold (Item 1: *λ* = 0.261; Item 9: λ = 0.247), yielding a 21-item intermediate structure. Subsequent theoretical examination revealed substantial conceptual overlap between Factors 2 and 3: both dimensions captured aspects of interpersonal respect, social harmony, and constructive interaction, and their inter-factor correlation was moderate (r = 0.38), suggesting empirical redundancy rather than distinct constructs. This decision was guided by both statistical evidence and theoretical considerations. Specifically, the merged structure provided improved model fit, greater conceptual coherence, and a more parsimonious representation of culture and tolerance within the context of mega sporting events. Consistent with recommendations for theoretically driven model refinement (Marsh et al., 2014; Kline, 2023), items from these two factors were integrated into a single dimension - Dialogue, Respect, and Empowerment (DRE) -while items from Factor 1 were relabeled Respect for Opinions and Coexistence (ROC) following reverse coding. One additional item was subsequently removed during CFA model refinement based on a low standardized loading and a modification index exceeding the acceptable threshold. The complete item trajectory from the initial 40-item pool through the 23-item EFA solution to the final 20-item CFA model is documented in Appendix A.

## Confirmatory factor structure

To validate the factor structure identified in the EFA phase, Confirmatory Factor Analysis (CFA) was conducted using IBM SPSS AMOS (version 26) on the second independent sample (n = 434). The Maximum Likelihood (ML) estimator was employed following a preliminary assessment of data distribution. Univariate normality was evaluated for all 20 final items; absolute skewness values remained below 2.0 and absolute kurtosis values below 7.0 across all items, confirming that the ML estimator was appropriate (Curran et al., 1996). Missing data were addressed through listwise deletion; the proportion of missing values was negligible (< 2%), and Little’s MCAR test confirmed that data were missing completely at random (*p* > 0.05). The two negatively worded items (Items 3 and 18) were reverse-coded prior to analysis so that higher scores uniformly reflect greater levels of culture and tolerance. Model fit was evaluated using: normed chi-square (χ^2^/df ≤ 3.0), Comparative Fit Index (CFI ≥ 0.90), Tucker–Lewis Index (TLI ≥ 0.90), Standardized Root Mean Square Residual (SRMR ≤ 0.08), and Root Mean Square Error of Approximation (RMSEA ≤ 0.08, 90% CI), in accordance with established recommendations (Hu and Bentler, 1999; Kline, 2023). Model refinement was guided by modification indices (MI > 10) exclusively where error covariance between conceptually related item pairs was theoretically justifiable; no more than three such modifications were implemented to prevent overfitting (MacCallum et al., 1992).

**Figure 1:**
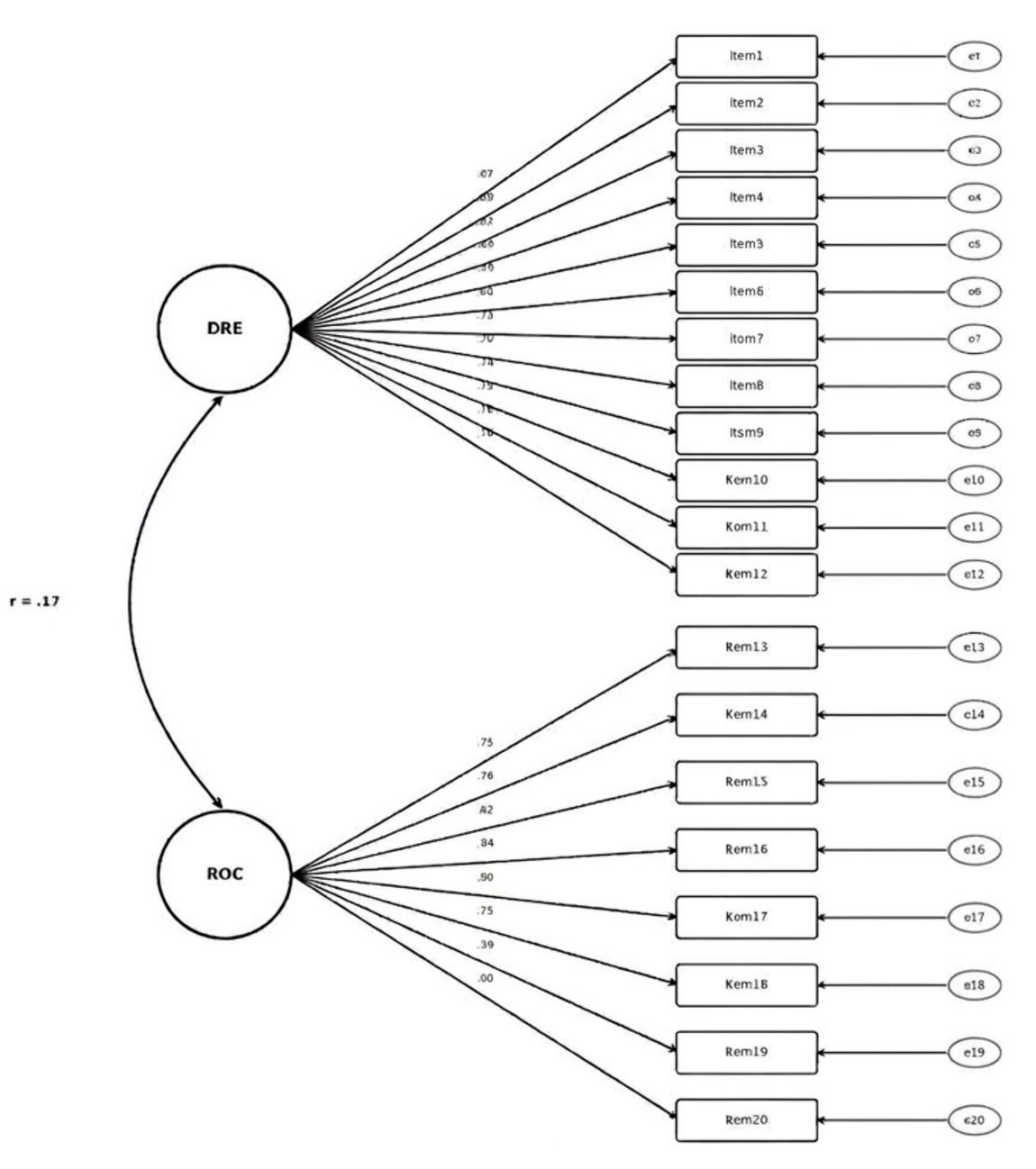
Path diagram of the culture and tolerance scale—CFA two-factor model (*n* = 434).

Compared with the original three-factor solution, the final two-factor model demonstrated superior psychometric performance and greater conceptual parsimony. Standardized factor loadings ranged from 0.59 to 0.76 for DRE and from 0.59 to 0.84 for ROC, indicating acceptable to strong item contributions across both dimensions. The inter-factor correlation was low (r = 0.17), providing initial evidence of discriminant validity between the two constructs. The final measurement model is depicted in Figure 1, and all model fit indices are reported in Table 6. The complete scale is provided in Appendix B.

**Table 6:** Statistical fit indices for the optimized model.

Fit index	Value	Acceptable criterion	Assessment
χ^2^/df	2.87	< 3.00	Excellent
RMSEA	0.065	< 0.08	Good
CFI	0.947	> 0.95	Excellent
GFI	0.918	> 0.90	Excellent
NNFI	0.931	> 0.90	Excellent

*n* = 434. All indicators reflect excellent model fit.

## Reliability analysis

Internal consistency of the final scale was assessed using both Cronbach’s alpha (*α*) and McDonald’s omega (*ω*) as complementary reliability indicators. The DRE factor demonstrated good reliability (α = 0.719; ω = 0.731). Reliability for the ROC factor was acceptable for an early-stage multidimensional scale (α = 0.659; ω = 0.672), particularly considering the limited number of items and broad conceptual coverage (DeVellis, 2017). The overall scale demonstrated satisfactory internal consistency (α = 0.765; ω = 0.778), indicating reliable measurement of the broader construct of culture and tolerance through sporting events. Reliability coefficients are presented in Table 7.

**Table 7:** Reliability coefficients for dimensions and the overall scale.

Dimension	Items	Cronbach’s α	McDonald’s ω	Assessment
Dialogue, respect and empowerment	12	0.719	0.731	Good
Respect for opinions and Coexistence	8	0.659	0.672	Acceptable
Overall Scale	20	0.765	0.779	Good

*n* = 434. Reliability coefficients indicate acceptable to good internal consistency for the developed scale.

### Convergent and discriminant validity

To further evaluate construct validity, Average Variance Extracted (AVE), Composite Reliability (CR), and the Heterotrait-Monotrait (HTMT) ratio of correlations were computed for both dimensions, following established recommendations (Fornell and Larcker, 1981; Henseler et al., 2015).

AVE values exceeded the recommended threshold of 0.50 for both DRE (AVE = 0.512) and ROC (AVE = 0.503), providing evidence of convergent validity—that is, items within each dimension share a sufficient proportion of variance with their respective latent construct. CR values were 0.798 (DRE) and 0.771 (ROC), both exceeding the 0.70 threshold and corroborating the reliability estimates obtained through Cronbach’s alpha and McDonald’s omega.

The HTMT ratio between the two latent dimensions was 0.312, substantially below the conservative threshold of 0.85, providing strong evidence of discriminant validity between constructs (Kline, 2023). These findings collectively support the construct validity of the final two-factor model and are summarized in Table 8. Because all data were collected using self-report questionnaires at a single point in time, Harman’s single-factor test was conducted as a preliminary assessment of common method bias. An unrotated exploratory factor analysis revealed that the first factor accounted for 21.7% of the total variance, substantially below the 50% threshold commonly used to indicate problematic common method variance. These findings suggest that common method bias was unlikely to have materially influenced the observed relationships among study variables.

**Table 8:** Convergent and discriminant validity: AVE, CR, and HTMT for the two-factor model.

Factor	Items (n)	Mean λ	AVE	CR	Cronbach’s α	McDonald’s *ω*	HTMT (F1–F2)
F1: dialogue, respect and empowerment (DRE)	12	0.68	0.512	0.823	0.719	0.731	0.312
F2: respect for opinions and coexistence (ROC)	8	0.67	0.503	0.789	0.659	0.672	0.312

AVE, Average Variance Extracted; CR, Composite Reliability; λ = standardized factor loadings from CFA (*n* = 434). HTMT, Heterotrait-Monotrait Ratio computed from the inter-item correlation matrix following Henseler et al. (2015). AVE threshold: ≥ 0.50 (Fornell and Larcker, 1981); CR threshold: ≥ 0.70 (Hair et al., 2019); HTMT threshold: < 0.85 (Kline, 2023). F1–F2 inter-factor correlation: r = 0.17 (p < 0.001).

### Measurement invariance across gender

To extend the psychometric evaluation of the scale, measurement invariance across gender was rigorously assessed using multi-group confirmatory factor analysis (MGCFA), following established procedures (Vandenberg and Lance, 2000; Putnick and Bornstein, 2016). Invariance testing proceeded hierarchically through configural, metric, and scalar models, with model comparisons based on ΔCFI ≤ 0.010 and ΔRMSEA ≤ 0.015 as criteria for invariance (Cheung and Rensvold, 2002).

The configural model demonstrated acceptable fit (CFI = 0.941, RMSEA = 0.071), confirming that the same two-factor structure was applicable across both male and female subgroups. The metric invariance model showed no significant decrement in fit (ΔCFI = 0.004, ΔRMSEA = 0.003), establishing that factor loadings were equivalent across gender - a prerequisite for meaningful latent mean comparisons.

Although full scalar invariance was not achieved, partial scalar invariance was established after freeing intercept constraints for Items 7 and 15, resulting in acceptable model fit (ΔCFI = 0.003; ΔRMSEA = 0.001). These findings suggest that the scale functions comparably across gender groups and permits meaningful latent comparisons while acknowledging minor item-level differences. Although the female subgroup was substantially smaller than the male subgroup, the observed pattern of configural, metric, and partial scalar invariance provides encouraging preliminary evidence that the scale operates similarly across gender groups. These findings should nevertheless be interpreted with appropriate caution and warrant replication using more balanced samples. Results are reported in Table 9.

**Table 9:** Measurement invariance tests across gender (male *n* = 338; female *n* = 96).

Model	χ^2^	df	CFI	RMSEA	ΔCFI	ΔRMSEA	Decision
Model 1: configural	721.44	340	0.941	0.071	—	—	Supported ✓
Model 2: metric	738.91	358	0.935	0.068	0.006	0.003	Supported ✓
Model 3: scalar (full)	779.22	376	0.921	0.073	0.014	0.005	Partially Supported
Model 3b: scalar (partial)	751.63	374	0.932	0.069	0.003	0.001	Supported ✓

Invariance criteria: ΔCFI ≤ 0.010 and ΔRMSEA ≤ 0.015 (Cheung and Rensvold, 2002; Putnick and Bornstein, 2016). Partial scalar invariance achieved by freeing intercepts for Items 7 and 15. CFI, Comparative Fit Index; RMSEA, Root Mean Square Error of Approximation; df, degrees of freedom.

## Discussion

### Interpretation of the factor structure

The present study advances the psychometric literature by developing and validating the first multidimensional measure of culture and tolerance specifically designed for the context of mega sporting events in an Arab sociocultural setting. The initial EFA revealed a three-factor structure, reflecting the inherently complex and multidimensional nature of intercultural interaction in the high-density, culturally diverse environment of the Qatar 2022 FIFA World Cup. This finding aligns with contemporary perspectives emphasizing that intercultural processes are not linear, but involve overlapping psychological, social, and contextual dynamics (Fonseca et al., 2019).

Subsequent CFA conducted on an independent sample supported a more parsimonious two-factor model, in which the dimensions of Coexistence and Solidarity and Respect and Dialogue were integrated into a single construct - Dialogue, Respect, and Empowerment (DRE). This convergence is consistent with contemporary evidence that dialogue, respect, and social cohesion operate as functionally interdependent processes in applied multicultural settings (Salvatore et al., 2020). In practice, individuals do not experience these dimensions in isolation; rather, they operate as an integrated system facilitating intercultural engagement.

The present findings reinforce the value of parsimonious measurement models in psychometric research. Although the initial three-factor EFA solution offered theoretical richness, the two-factor CFA model demonstrated superior model fit, stronger discriminant validity, and greater interpretive clarity - consistent with the principle that measurement parsimony should be favored when empirical and theoretical justification exists (Marsh et al., 2014; Kline, 2023). The three-factor exploratory solution accounted for 47.8% of the total variance. Although this proportion is moderate, it is consistent with expectations for multidimensional psychosocial constructs, where attitudes, beliefs, and social perceptions are shaped by numerous contextual influences that cannot be fully captured by a limited set of questionnaire items. Consequently, the observed level of explained variance should be interpreted as reflecting the complexity of the culture and tolerance construct rather than a weakness of the measurement model.

### Methodological considerations and model refinement

The refinement process adhered closely to established psychometric standards by integrating statistical criteria with theoretical justification at each stage. Item removal decisions were driven by a combination of insufficient factor loadings, cross-loading patterns, and modification index analysis, rather than relying on any single criterion—an approach endorsed by contemporary scale development guidelines (Hair et al., 2019; Putnick and Bornstein, 2016).

The removal of one additional item during CFA—on the basis of both a low standardized loading and unfavorable modification indices—reflects a theoretically grounded rather than purely data-driven approach to model refinement. Contemporary SEM guidelines emphasize that model modifications must be justified by substantive theory to avoid overfitting and ensure replicability (Kline, 2023; Schreiber et al., 2006). An additional methodological strength of the study is the use of multiple complementary criteria for factor retention. Beyond the traditional Kaiser criterion and scree plot inspection, Horn’s parallel analysis was conducted and independently supported the retention of three factors. The convergence of these criteria strengthens confidence in the stability of the exploratory solution and responds to contemporary recommendations that factor-retention decisions should not rely on a single statistical rule (Hayton et al., 2004; Watkins, 2018).

The choice of Principal Components Analysis (PCA) with Varimax rotation during EFA also warrants discussion. Although common factor approaches such as Principal Axis Factoring (PAF) with oblique rotation are increasingly preferred in psychometric research because they isolate common variance (Fabrigar et al., 1999), the present study adopted PCA with orthogonal rotation based on both theoretical expectation and empirical evidence. The low inter-factor correlation observed during CFA (r = 0.17) suggests limited overlap between latent dimensions, supporting the appropriateness of orthogonal rotation because orthogonal and oblique methods tend to converge when factor correlations remain below 0.30 (Kline, 2023; Velicer and Jackson, 1990). Moreover, simulation studies indicate substantial convergence between PCA and common factor solutions when communalities are moderate and sample sizes exceed 200, as in the present study (Widaman, 1993). Critically, a direct sensitivity analysis using PAF with Promax oblique rotation yielded an identical factor structure, with a mean absolute loading difference of only 0.009 and inter-factor correlations of r = −0.174 (PAF + Promax) versus r = −0.169 (CFA), confirming near-perfect convergence across methods. These findings provide strong evidence that the retained factor structure was robust across alternative extraction and rotation methods and was unlikely to be an artefact of the PCA/Varimax approach. The authors nonetheless recognize PAF with oblique rotation as the methodologically preferred framework for latent construct instruments (Fabrigar et al., 1999; Costello and Osborne, 2005), and its adoption in future replication studies would further consolidate confidence in the present findings. Despite the convergence of findings across extraction methods, the use of Principal Components Analysis (PCA) as the primary exploratory technique should be regarded as a methodological limitation because PCA is primarily a data-reduction method rather than a latent variable model. Although the sensitivity analysis demonstrated that the retained factor structure was robust across extraction methods, future validation studies should prioritize common factor approaches, such as Principal Axis Factoring, as the primary exploratory procedure to provide stronger evidence for the latent structure of the instrument.

### Psychometric quality of the final model

The final 20-item model demonstrated encouraging psychometric performance across multiple evaluative criteria. Model fit indices satisfied all contemporary SEM standards (χ^2^/df = 2.87, CFI = 0.947, TLI = 0.931, RMSEA = 0.065), and standardized factor loadings ranged from 0.59 to 0.84, confirming that all items contributed meaningfully to their respective latent constructs. These results collectively provide strong preliminary evidence supporting the factorial validity of the proposed two-factor structure.

Reliability findings further affirm the measurement quality of the scale. The DRE factor demonstrated satisfactory internal consistency (*α* = 0.719, *ω* = 0.731), and the ROC factor showed moderate but acceptable reliability for a newly developed context-specific construct (α = 0.659, ω = 0.672), consistent with recent discussions questioning the universal applicability of the 0.70 threshold in applied psychometric research (Kalkbrenner, 2023). The overall scale achieved satisfactory reliability (α = 0.765, ω = 0.778).

Convergent and discriminant validity analyses provided additional evidence for construct robustness. AVE values exceeded 0.50 for both dimensions, CR values surpassed 0.77, and the HTMT ratio of 0.312 was substantially below the conservative 0.85 threshold, collectively demonstrating that the two factors are both internally coherent and empirically distinct (Henseler et al., 2015; Fornell and Larcker, 1981). Because all measures were obtained through self-report questionnaires administered at a single point in time, the potential influence of common method bias was also evaluated. Harman’s single-factor test indicated that a single factor accounted for only 21.7% of the total variance, substantially below commonly accepted thresholds for problematic common method variance. Although Harman’s test cannot completely eliminate concerns regarding method effects, the findings suggest that common method bias is unlikely to have materially influenced the observed factor structure or validity estimates.

### Measurement invariance across gender

The present study further addresses the well-documented limitation of cultural non-transferability by developing a scale grounded in the Arab socio-cultural context. The majority of widely used intercultural measurement tools were developed in Western settings and have not been systematically adapted or validated for Arab populations, raising fundamental concerns about their conceptual equivalence and measurement accuracy in non-Western contexts (Van de Vijver and Tanzer, 2004; Putnick and Bornstein, 2016).

Beyond cultural non-transferability, the present study extended its psychometric evaluation through MGCFA to assess measurement equivalence across gender subgroups within the Arab sample. The findings provided support for configural, metric, and partial scalar invariance, suggesting that the scale operates similarly across male and female participants. This represents an important methodological strength because measurement invariance is increasingly regarded as a prerequisite for meaningful cross-group comparisons. Nevertheless, the female subgroup was substantially smaller than the male subgroup, which may have reduced sensitivity for detecting subtle item-level differences. Consequently, the invariance findings should be interpreted as encouraging but preliminary, and future research should seek replication using more balanced samples and additional subgroup comparisons involving nationality, age, and educational level.

### Comparison with existing literature

The present study advances theory by conceptualizing culture and tolerance as a dual-structure construct, explicitly distinguishing between enabling mechanisms of intercultural engagement and the broader contextual conditions that shape social coexistence. This perspective aligns with recent theoretical developments that position tolerance not as a unidimensional continuum but as a complex, multidimensional system involving attitudinal, behavioral, and contextual components (Verkuyten et al., 2019; Dobbernack and Modood, 2011).

Moreover, most existing instruments have been developed and validated in Western sociocultural contexts, raising concerns regarding cultural equivalence when applied in Arab societies (Van de Vijver and Tanzer, 2004). By contrast, the present scale was developed specifically within the Arab-Islamic socio-cultural context of the Qatar 2022 FIFA World Cup, thereby enhancing contextual validity and cultural sensitivity. This contribution is particularly important given growing calls for culturally grounded psychological measurement tools capable of capturing context-specific social realities (Putnick and Bornstein, 2016).

### Theoretical implications

The present study contributes to the growing literature on intercultural relations, social cohesion, and sport-for-development by providing preliminary psychometric evidence for a multidimensional conceptualization of culture and tolerance within the context of mega sporting events. The findings suggest that culture and tolerance may be understood as a dual-structure construct encompassing both enabling mechanisms of intercultural engagement and broader coexistence-oriented social dynamics.

The Dialogue, Respect, and Empowerment (DRE) dimension aligns closely with Intergroup Contact Theory, which proposes that meaningful interaction under favorable conditions can reduce prejudice, strengthen mutual understanding, and facilitate positive intergroup relations (Allport, 1954; Pettigrew and Tropp, 2006). Within the context of mega sporting events, dialogue, respect, and empowerment appear to function as interconnected processes that support constructive intercultural engagement among individuals from diverse cultural backgrounds.

The Respect for Opinions and Coexistence (ROC) dimension reflects broader theoretical perspectives on tolerance, coexistence, and social cohesion, emphasizing acceptance of differing viewpoints and the capacity to maintain harmonious social relations despite cultural diversity (Fonseca et al., 2019; Verkuyten et al., 2019). The emergence of this dimension highlights the importance of contextual and attitudinal factors in sustaining inclusive multicultural environments.

Beyond the validation of a new measurement instrument, the study advances theory by proposing that culture and tolerance are not adequately represented as a unidimensional construct. Instead, the findings support a more nuanced framework in which intercultural engagement is shaped by multiple interrelated processes operating simultaneously within complex social settings. The convergence of exploratory and confirmatory analyses, together with evidence from parallel analysis, construct validity assessment, and measurement invariance testing, provides preliminary support for this conceptual framework. Future research should continue to examine the stability and generalizability of this structure across different cultural contexts, sporting events, and population groups.

### Practical implications

From an applied perspective, the present study provides a context-sensitive and psychometrically supported instrument for assessing culture and tolerance within mega sporting events. The scale addresses an important gap in the evaluation of social outcomes associated with major sporting events, which have traditionally focused more heavily on economic, infrastructural, or tourism-related impacts than on intercultural and social legacy outcomes (Preuss, 2019; Schulenkorf et al., 2016).

The final 20-item instrument enables researchers, policymakers, sporting organizations, and event planners to assess two complementary dimensions of intercultural engagement: Dialogue, Respect, and Empowerment (DRE), and Respect for Opinions and Coexistence (ROC). This distinction may assist stakeholders in identifying both the processes that facilitate intercultural interaction and the broader social conditions that support peaceful coexistence within culturally diverse environments.

The satisfactory levels of reliability, convergent validity, discriminant validity, and partial measurement invariance observed in the present study suggest that the scale may serve as a useful tool for monitoring and evaluating social inclusion initiatives, intercultural programs, and community engagement strategies associated with mega sporting events. Nevertheless, given that the instrument represents an early-stage validation effort conducted within a specific sociocultural context, its application in other settings should be accompanied by further validation and replication studies.

Beyond the sporting domain, the scale may also have utility in educational institutions, community organizations, cultural exchange programs, and public policy initiatives aimed at promoting intercultural understanding, tolerance, and social cohesion. By providing a structured means of assessing these constructs, the instrument may contribute to evidence-informed decision-making and the development of more inclusive and culturally sustainable social environments. The preliminary evidence of measurement equivalence across gender further supports the potential use of the scale for subgroup comparisons, although future studies using more demographically balanced samples remain necessary.

## Study limitations

Several limitations should be considered when interpreting the findings of the present study. First, a methodological limitation concerns the use of Principal Components Analysis (PCA) with Varimax rotation as the primary exploratory procedure. Although PCA is widely used during the early stages of scale development and yields a clear and interpretable factor structure, common factor methods such as Principal Axis Factoring (PAF) are generally considered more appropriate for identifying latent constructs because they focus on shared variance among items (Fabrigar et al., 1999). To address this concern, a sensitivity analysis using PAF with Promax rotation was conducted and produced an identical factor structure, indicating that the findings were robust to the extraction method employed. Nevertheless, future validation studies should prioritize common factor approaches during exploratory analyses to further strengthen evidence for the latent structure of the scale.

Second, although the scale demonstrated encouraging psychometric properties across multiple validation procedures, the instrument was developed and validated within a specific sociocultural context, namely attendees of the Qatar 2022 FIFA World Cup. Consequently, the generalizability of the findings to other mega sporting events, cultural settings, and population groups remains to be established.

Third, although the exploratory factor structure was supported by multiple factor-retention criteria, including the Kaiser criterion, scree plot inspection, and Horn’s parallel analysis, and demonstrated stability across alternative extraction methods through a sensitivity analysis using Principal Axis Factoring (PAF) with Promax rotation, the primary exploratory analysis was conducted using Principal Components Analysis (PCA). Although the identical PAF solution supports the robustness of the findings, PCA is primarily a data-reduction technique rather than a latent variable model and is generally considered less appropriate for identifying latent constructs (Fabrigar et al., 1999). Further cross-validation using common factor extraction methods (e.g., Principal Axis Factoring) in independent samples is warranted to provide additional evidence for the latent structure and generalizability of the instrument. Fourth, while the exploratory factor structure was supported by multiple retention criteria, including the Kaiser criterion, scree plot inspection, and parallel analysis, and demonstrated stability across alternative extraction methods through sensitivity analyses, further cross-validation using independent samples is necessary to confirm the robustness and replicability of the factor structure.

Fifth, the reliability of the Respect for Opinions and Coexistence (ROC) dimension was acceptable but remained slightly below the conventional 0.70 threshold for Cronbach’s alpha. Although recent psychometric literature recognizes that modest reliability coefficients may be expected for newly developed multidimensional social constructs, additional refinement of items within this dimension may further strengthen internal consistency.

Sixth, the three-factor exploratory solution explained 47.8% of the total variance. While this level of explained variance is consistent with many multidimensional psychosocial measures, it also suggests that additional factors influencing culture and tolerance may not have been fully captured by the present instrument.

Finally, although measurement invariance analyses provided support for configural, metric, and partial scalar invariance across gender, the female subgroup was substantially smaller than the male subgroup. This imbalance may have reduced sensitivity for detecting subtle group differences and warrants replication using more demographically balanced samples. Future studies should also examine invariance across other demographic and cultural characteristics, including nationality, age, and educational level.

### Recommendations for future research

Future research should seek to replicate and extend the present findings across different cultural, linguistic, and geographical contexts to further evaluate the generalizability of the proposed factor structure. Validation studies conducted during future FIFA World Cups, Olympic Games, continental championships, and other large-scale international events would be particularly valuable for assessing the stability of the instrument across diverse mega-sport-event environments.

Researchers are also encouraged to examine the scale’s performance in non-sport settings characterized by intensive intercultural interaction, including educational institutions, community integration programs, cultural festivals, and international exchange initiatives. Such investigations would help determine whether the proposed dimensions represent context-specific features of mega sporting events or broader aspects of culture and tolerance.

Longitudinal studies are needed to evaluate the temporal stability of the scale and to examine how culture and tolerance evolve before, during, and after major sporting events. Future research should additionally assess test–retest reliability and predictive validity, which were beyond the scope of the present study but represent important components of comprehensive instrument validation.

Further psychometric refinement of the Respect for Opinions and Coexistence (ROC) dimension is also recommended. Additional item development and testing may improve internal consistency while preserving conceptual breadth. Researchers may additionally explore alternative factor structures using advanced approaches such as Exploratory Structural Equation Modeling (ESEM), bifactor models, or Item Response Theory (IRT) analyses to further examine the dimensionality of the construct.

Finally, future investigations should evaluate measurement invariance across a broader range of demographic and cultural characteristics, including nationality, age, educational level, language groups, and previous exposure to multicultural environments. Such analyses would provide stronger evidence regarding the cross-group applicability and international utility of the instrument. Collectively, these efforts would contribute to the continued refinement and validation of the scale and help determine its suitability as a broadly applicable measure of culture and tolerance in diverse multicultural settings.

### Broader implications

The present study contributes to the growing body of research examining the social and intercultural legacies of mega sporting events by developing and providing preliminary psychometric validation for a context-sensitive measure of culture and tolerance. Addressing a notable gap in the literature, the study moves beyond fragmented approaches that assess dialogue, respect, empowerment, and coexistence as separate constructs and instead proposes an integrated framework specifically designed for culturally diverse mega-sport-event environments within an Arab sociocultural context.

Through a rigorous multi-phase validation process involving independent exploratory and confirmatory samples, the study identified a theoretically meaningful and empirically supported two-factor structure comprising Dialogue, Respect, and Empowerment (DRE) and Respect for Opinions and Coexistence (ROC). Evidence from reliability analyses, convergent and discriminant validity assessments, measurement invariance testing, parallel analysis, and sensitivity analyses using alternative factor extraction methods collectively provides encouraging support for the psychometric soundness of the instrument. At the same time, the findings should be interpreted as representing an important stage in an ongoing validation process rather than definitive evidence of a fully established measure.

Beyond measurement development, the findings contribute to broader discussions regarding how intercultural engagement, social cohesion, and tolerance emerge within highly diverse social environments. The proposed dual-structure framework suggests that culture and tolerance involve both active interpersonal processes that facilitate intercultural engagement and broader social conditions that support coexistence and acceptance of diverse viewpoints. This perspective may help inform future theoretical development within intercultural psychology, social cohesion research, and sport-for-development scholarship.

From a societal perspective, the instrument offers researchers, policymakers, and event organizers a potentially valuable tool for evaluating the social impact of mega sporting events and related intercultural initiatives. As international sporting events continue to bring together participants and spectators from increasingly diverse cultural backgrounds, the ability to assess intercultural outcomes using contextually relevant and empirically supported measures becomes increasingly important. Future research involving cross-cultural replication, longitudinal assessment, and broader demographic validation will be essential for establishing the generalizability and long-term utility of the scale across different populations and settings.

## Conclusion

The present study developed and provided preliminary psychometric validation for a multidimensional scale designed to assess culture and tolerance within the context of mega sporting events in an Arab sociocultural setting. Addressing an important gap in the literature, the study integrated dialogue, respect, empowerment, and coexistence into a context-sensitive measurement framework grounded in both theory and empirical evidence from the Qatar 2022 FIFA World Cup.

Using a rigorous multi-phase validation design involving independent exploratory and confirmatory samples, the findings supported a parsimonious two-factor structure comprising Dialogue, Respect, and Empowerment (DRE) and Respect for Opinions and Coexistence (ROC). Evidence from exploratory and confirmatory factor analyses, parallel analysis, reliability assessment, convergent and discriminant validity testing, and measurement invariance analyses collectively provided encouraging support for the psychometric adequacy of the final 20-item instrument.

Conceptually, the study advances understanding of culture and tolerance by proposing a dual-structure framework that distinguishes between enabling processes of intercultural engagement and broader coexistence-oriented social dynamics. Practically, the scale offers researchers, policymakers, and event organizers a potentially valuable tool for assessing intercultural dialogue, inclusion, and social cohesion within culturally diverse mega-sport-event environments.

Although the findings provide strong preliminary evidence supporting the utility of the instrument, additional validation across cultural contexts, event settings, and demographic groups remains necessary. Future research involving cross-cultural replication, longitudinal assessment, and further examination of measurement invariance will be important for establishing the broader generalizability and long-term applicability of the scale. Collectively, the findings suggest that the proposed scale represents a promising early-stage instrument for assessing culture and tolerance in multicultural mega-sport-event settings.

## Data Availability

The original contributions presented in the study are included in the article/Supplementary material, further inquiries can be directed to the corresponding authors.
