# Supplementary material for: Development and psychometric validation of a culture and tolerance scale in mega sporting events: evidence from an Arab context
Source: Front Psychol. 2026 Aug 5;17:1887691. doi: 10.3389/fpsyg.2026.1887691 (PMC13485874; doi:10.3389/fpsyg.2026.1887691)
Supplement: Supplementary file 1 [file Supplementary_File_1.docx]

**Appendix A. Complete Item Trajectory: From the 40-Item Pool to the Final 20-Item Scale**

The table below documents the full item development process across three stages: (1) the initial 40-item pool constructed from the literature review; (2) the 23-item intermediate pool retained after EFA (n = 246); and (3) the final 20-item scale after CFA refinement (n = 434). Items marked 'Negative' represent reverse-worded items and were recoded prior to analysis. Reasons for exclusion are provided for all removed items. The S-CVI/Ave for the expert-reviewed 40-item pool was .87, exceeding the recommended threshold of .80 (Polit & Beck, 2006).

| Original No. (Pool of 40) | EFA No. | Item Content *(During the Qatar 2022 World Cup…)* | Item Type | EFA Factor | EFA Loading | EFA Decision | CFA Decision | Final Scale No. | Factor Assignment |
| --- | --- | --- | --- | --- | --- | --- | --- | --- | --- |
| 1 | 1 | There were effective listening and respect for differing viewpoints. | Negative [R] | F3 | .261 | ❌ Removed — Low factor loading (λ = .261 < .40) | — | — | — |
| 2 | 2 | Opinions were expressed respectfully, even when there was disagreement. | Positive | F3 | .814 | ✅ Retained | ✅ Retained | **2** | DRE |
| 3 | 3 | Individuals had difficulty understanding others' different perspectives. | Negative [R] | F1 | .820 | ✅ Retained | ✅ Retained | **3** | DRE |
| 4 | 4 | Everyone sought consensus solutions when disagreements arose. | Positive | F3 | .553 | ✅ Retained | ✅ Retained | **4** | DRE |
| 5 | 5 | Individuals actively participated in dialogues with people from different backgrounds. | Positive | F1 | .694 | ✅ Retained | ✅ Retained | **5** | DRE |
| 6 | 6 | Everyone recognized the importance of constructive dialogue in solving problems. | Positive | F3 | .672 | ✅ Retained | ✅ Retained | **6** | DRE |
| 7 | 7 (9) | Individuals respected the values and customs of others even when they differed. | Positive | F1 | .595 | ✅ Retained | ✅ Retained | **7** | DRE |
| 8 | 8 (11) | Everyone accepted religious and cultural diversity in society. | Positive | F1 | .683 | ✅ Retained | ✅ Retained | **8** | DRE |
| 9 | 9 (13) | Others respected and valued individuals' opinions. | Positive | F3 | .247 | ❌ Removed — Low factor loading (λ = .247 < .40) | — | — | — |
| 10 | 10 (14) | Individuals treated others with respect and dignity regardless of their backgrounds. | Positive | F2 | .784 | ✅ Retained | ✅ Retained | **9** | DRE |
| 11 | 11 (18) | Individuals had influence in important societal decisions. | Positive | F1 | .735 | ✅ Retained | ✅ Retained | **10** | DRE |
| 12 | 12 (20) | Individuals possessed the power and ability to make a positive impact on their surroundings. | Positive | F1 | .700 | ✅ Retained | ✅ Retained | **11** | DRE |
| 13 | 13 (22) | There were effective listening and respect for others' differing viewpoints. | Positive | F1 | .744 | ✅ Retained | ✅ Retained | **1** | DRE |
| 14 | 14 (24) | Everyone actively participated in community projects and activities. | Positive | F1 | .752 | ✅ Retained | ✅ Retained | **12** | DRE |
| 15 | 15 (26) | Individuals felt a sense of responsibility toward community well-being. | Positive | F2 | .844 | ✅ Retained | ✅ Retained | **13** | ROC |
| 16 | 16 (28) | Individuals possessed the skills and resources needed to achieve their goals. | Positive | F2 | .824 | ✅ Retained | ✅ Retained | **14** | ROC |
| 17 | 17 (30) | Everyone lived in peace with people from diverse backgrounds. | Positive | F1 | .782 | ✅ Retained | ✅ Retained | **15** | ROC |
| 18 | 18 (32) | Everyone preferred to resolve conflicts through peaceful and civilized means. | Positive | F1 | .800 | ✅ Retained | ✅ Retained | **16** | ROC |
| 19 | 19 (34) | Diversity threatened societal stability and cohesion. | Negative [R] | F1 | .750 | ✅ Retained | ✅ Retained | **17** | ROC |
| 20 | 20 (36) | Everyone contributed to spreading a culture of tolerance and peaceful coexistence. | Positive | F1 | .764 | ✅ Retained | ✅ Retained | **18** | ROC |
| 21 | 21 (38) | Individuals felt psychological and social safety in the community. | Positive | F2 | .589 | ✅ Retained | ✅ Retained | **19** | ROC |
| **22** | **22 (39)** | **Everyone actively participated in community projects and activities.** *(duplicate of EFA Item 14)* | Positive | F2 | .602 | ✅ Retained | ❌ **Removed** — Conceptual redundancy with Item 14; low standardized loading; modification index > 10 | **—** | — |
| 23 | 23 (40) | Individuals felt a sense of responsibility toward community well-being. | Positive | F1 | .711 | ✅ Retained | ✅ Retained | **20** | ROC |
| 24–40 | — | *(Items 24–40: various items on teamwork, empowerment, inclusion, and cultural heritage)* | Mixed | — | — | ❌ Removed — I-CVI < .75 (content validity stage) | — | — | — |

**Note.** [R] = reverse-scored item; scores recoded prior to analysis so that higher values reflect greater culture and tolerance. — = item did not reach this stage. Item wording in this appendix corresponds exactly to Appendix B in the manuscript. DRE = Dialogue, Respect & Empowerment (12 items, Final Nos. 1–12); ROC = Respect for Opinions & Coexistence (8 items, Final Nos. 13–20). EFA removal criteria: factor loading < .40 or cross-loading > .30. CFA removal criteria: low standardized loading combined with modification index > 10.
