# Supplementary material for: Development and psychometric validation of a culture and tolerance scale in mega sporting events: evidence from an Arab context
Source: Front Psychol. 2026 Aug 5;17:1887691. doi: 10.3389/fpsyg.2026.1887691 (PMC13485874; doi:10.3389/fpsyg.2026.1887691)
Supplement: Supplementary file 2 [file Supplementary_File_2.docx]

**Appendix B. Culture and Tolerance Through Sporting Events Scale — Final Version (20 Items)**

Instructions: Dear participant, please answer the following items honestly based on your experience attending Qatar 2022 FIFA World Cup events. All your answers are confidential and used for scientific research purposes only. Please select the response that best expresses your opinion on a scale from 1 to 5.

| **#** | **Item (During the Qatar 2022 World Cup…)** | **Type** | **1 Strongly Disagree** | **2 Disagree** | **3 Neutral** | **4 Agree** | **5 Strongly Agree** |
| --- | --- | --- | --- | --- | --- | --- | --- |
| **#** | **Dimension one: Dialogue, Respect, and Empowerment (12 Items)** | | | | | | |
| 1 | There were effective listening and respect for others' differing viewpoints. | Positive | ☐ | ☐ | ☐ | ☐ | ☐ |
| 2 | Opinions were expressed respectfully, even when there was disagreement. | Positive | ☐ | ☐ | ☐ | ☐ | ☐ |
| 3 | Individuals had difficulty understanding others' different perspectives. | Negative | ☐ | ☐ | ☐ | ☐ | ☐ |
| 4 | Everyone sought consensus solutions when disagreements arose. | Positive | ☐ | ☐ | ☐ | ☐ | ☐ |
| 5 | Individuals actively participated in dialogues with people from different backgrounds. | Positive | ☐ | ☐ | ☐ | ☐ | ☐ |
| 6 | Everyone recognized the importance of constructive dialogue in solving problems. | Positive | ☐ | ☐ | ☐ | ☐ | ☐ |
| 7 | Individuals respected the values and customs of others even when they differed. | Positive | ☐ | ☐ | ☐ | ☐ | ☐ |
| 8 | Everyone accepted religious and cultural diversity in society. | Positive | ☐ | ☐ | ☐ | ☐ | ☐ |
| 9 | Individuals treated others with respect and dignity regardless of their backgrounds. | Positive | ☐ | ☐ | ☐ | ☐ | ☐ |
| 10 | Individuals had influence in important societal decisions. | Positive | ☐ | ☐ | ☐ | ☐ | ☐ |
| 11 | Individuals possessed the power and ability to make a positive impact on their surroundings. | Positive | ☐ | ☐ | ☐ | ☐ | ☐ |
| 12 | Everyone actively participated in community projects and activities. | Positive | ☐ | ☐ | ☐ | ☐ | ☐ |
| # | **Dimension two: Respect for Opinions and Coexistence (8 Items)** | | | | | | |
| 13 | Individuals felt a sense of responsibility toward community well-being. | Positive | ☐ | ☐ | ☐ | ☐ | ☐ |
| 14 | Individuals possessed the skills and resources needed to achieve their goals. | Positive | ☐ | ☐ | ☐ | ☐ | ☐ |
| 15 | Everyone lived in peace with people from diverse backgrounds. | Positive | ☐ | ☐ | ☐ | ☐ | ☐ |
| 16 | Everyone preferred to resolve conflicts through peaceful and civilized means. | Positive | ☐ | ☐ | ☐ | ☐ | ☐ |
| 17 | Diversity threatened societal stability and cohesion. | Negative | ☐ | ☐ | ☐ | ☐ | ☐ |
| 18 | Everyone contributed to spreading a culture of tolerance and peaceful coexistence. | Positive | ☐ | ☐ | ☐ | ☐ | ☐ |
| 19 | Individuals felt psychological and social safety in the community. | Positive | ☐ | ☐ | ☐ | ☐ | ☐ |
| 20 | Individuals felt a sense of responsibility toward community well-being. | Positive | ☐ | ☐ | ☐ | ☐ | ☐ |

Note: Items marked “Negative” represent reverse-worded perspectives and must have their scores reversed during score.
